# Survivin’ Acute Myeloid Leukaemia—A Personalised Target for inv(16) Patients

**DOI:** 10.3390/ijms221910482

**Published:** 2021-09-28

**Authors:** Jochen Greiner, Elliott Brown, Lars Bullinger, Robert K. Hills, Vanessa Morris, Hartmut Döhner, Ken I. Mills, Barbara-ann Guinn

**Affiliations:** 1 Department of Internal Medicine, Diakonie Hospital Stuttgart, 70176 Stuttgart, Germany; greiner@diak-stuttgart.de; 2 Department of Internal Medicine III, University of Ulm, Helmholtzstr. 10, 89081 Ulm, Germany; hartmut.doehner@unikinik-ulm.de; 3 Department of Biomedical Sciences, University of Hull, Hull HU6 7RX, UK; Elliott.Brown-2016@hull.ac.uk (E.B.); V.S.Morris-2017@hull.ac.uk (V.M.); 4 Department of Hematology, Oncology and Tumor Immunology, Charité–Universitätsmedizin Berlin, 13353 Berlin, Germany; lars.bullinger@charite.de; 5 German Cancer Consortium (DKTK), Partner site Berlin, 13353 Berlin, Germany; 6 Nuffield Department of Population Health, Richard Doll Building, University of Oxford, Oxford OX3 7LF, UK; robert.hills@ndph.ox.ac.uk; 7 Patrick G. Johnson Centre for Cancer Research, Queen’s University Belfast, Lisburn Road, Belfast BT9 7AE, UK; K.Mills@qub.ac.uk

**Keywords:** BIRC5, overall survival, survivin, acute myeloid leukaemia, Core Binding Factor (CBF), inv(16)

## Abstract

Despite recent advances in therapies including immunotherapy, patients with acute myeloid leukaemia (AML) still experience relatively poor survival rates. The Inhibition of Apoptosis (IAP) family member, survivin, also known by its gene and protein name, Baculoviral IAP Repeat Containing 5 (BIRC5), remains one of the most frequently expressed antigens across AML subtypes. To better understand its potential to act as a target for immunotherapy and a biomarker for AML survival, we examined the protein and pathways that BIRC5 interacts with using the Kyoto Encyclopedia of Genes and Genomes (KEGG), search tool for recurring instances of neighbouring genes (STRING), WEB-based Gene Set Analysis Toolkit, Bloodspot and performed a comprehensive literature review. We then analysed data from gene expression studies. These included 312 AML samples in the Microarray Innovations In Leukemia (MILE) dataset. We found a trend between above median levels of BIRC5 being associated with improved overall survival (OS) but this did not reach statistical significance (*p* = 0.077, Log-Rank). There was some evidence of a beneficial effect in adjusted analyses where above median levels of BIRC5 were shown to be associated with improved OS (*p* = 0.001) including in Core Binding Factor (CBF) patients (*p* = 0.03). Above median levels of BIRC5 transcript were associated with improved relapse free survival (*p* < 0.0001). Utilisation of a second large cDNA microarray dataset including 306 AML cases, again showed no correlation between BIRC5 levels and OS, but high expression levels of BIRC5 correlated with worse survival in inv(16) patients (*p* = 0.077) which was highly significant when datasets A and B were combined (*p* = 0.001). In addition, decreased BIRC5 expression was associated with better clinical outcome (*p* = 0.004) in AML patients exhibiting CBF mainly due to patients with inv(16) (*p* = 0.007). This study has shown that BIRC5 expression plays a role in the survival of AML patients, this association is not apparent when we examine CBF patients as a cohort, but when those with inv(16) independently indicating that those patients with inv(16) would provide interesting candidates for immunotherapies that target BIRC5.

## 1. Introduction

Acute Myeloid Leukaemia (AML) is defined as a malignant disorder of the bone marrow (BM) characterised by the clonal expansion and differentiation arrest of myeloid progenitor cells [1]. AML is difficult to treat, mostly due to its heterogeneity and the older age group it arises in. AML is now diagnosed in accordance with the World Health Organisation (WHO) criteria, which was revised in 2016 to integrate new methods of diagnosis such as updates in genetic data, biomarkers, morphology and immunotherapy [2]. Patient outcomes can be predicted by the cytogenetic abnormalities detected in their blasts with t(8;21), t(15;17), inv(16), and t(16;16) or biallelic CCAAT/enhancer-binding protein alpha (CEBPA), a transcription factor that controls proliferation and granulocytic differentiation, all being associated with favourable prognosis (60% overall survival (OS) and 90% remission rate) [3,4]. Poor prognostic markers include inv(3), t(3;3), t(6;9), −5, 5q-, −7, 7q- or complex karyotype as these patients are highly resistant to induction chemotherapy, have higher relapse rates and an OS of just 5–15% [5].

Despite treatments such as maximally intensive chemotherapies and allogeneic stem cell transplantation, survival rates have remained mostly unchanged for AML patients until recent years when there has been a significant shift towards the use of novel and effective, targeted therapies including inhibitors of mutant FMS-like tyrosine kinase 3 (FLT3) [6] and isocitrate dehydrogenase (IDH), the B cell lymphoma 2 inhibitor venetoclax and the hedgehog pathway inhibitor glasdegib (reviewed in [7]). Although unique cytogenetic abnormalities occur in many AML patients, most account for less than 10% of all patients and few have been found to be suitable targets for therapy with few exceptions [8].

Baculoviral IAP Repeat Containing 5 (BIRC5) is expressed in 60% of adult AML patient samples and is more frequently expressed than FLT-3 [9], PRAME [10] or Wilms’ Tumour gene 1 (WT1) [11]. It is an apoptosis inhibitor [12] normally found in embryonic development and absent from normal differentiated tissues. BIRC5 is commonly upregulated within tumours [13] and its overexpression is associated with a worse prognosis in a number of different cancer types [14,15,16], likely due to a failure of programmed cell death in the affected cells. BIRC5 plays an essential role in mitosis and secures bipolar chromosome segregation with its molecular partners, Aurora B, Borealin and the inner centromere protein, playing a key role in chromosomal instability when overexpressed [17]. BIRC5 has been shown to be transcriptionally repressed by wild-type p53 [18] and when p53 is absent or mutated, BIRC5 overexpression leads to polyploidy.

In 2020, Davis et al. [19] described the identification of genes that were differentially expressed between adult AML risk subgroups following analysis of The Cancer Genome Atlas (TCGA-LAML) dataset. Only risk subgroups that included more than 10 patients were reported on. We found that genes altered in AML were involved in key processes such as the evasion of apoptosis (*BIRC5*, *WNT1*) or the control of cell proliferation (*SSX2IP*, *AML1-ETO*). On this basis, and its relatively high frequency of expression in AML, we examined BIRC5, its molecular interactions, its potential as a biomarker and target for therapy in AML, further. 

## 2. Results

### 2.1. BIRC5 Expression in Healthy Blood Cells

**Figure 1:**
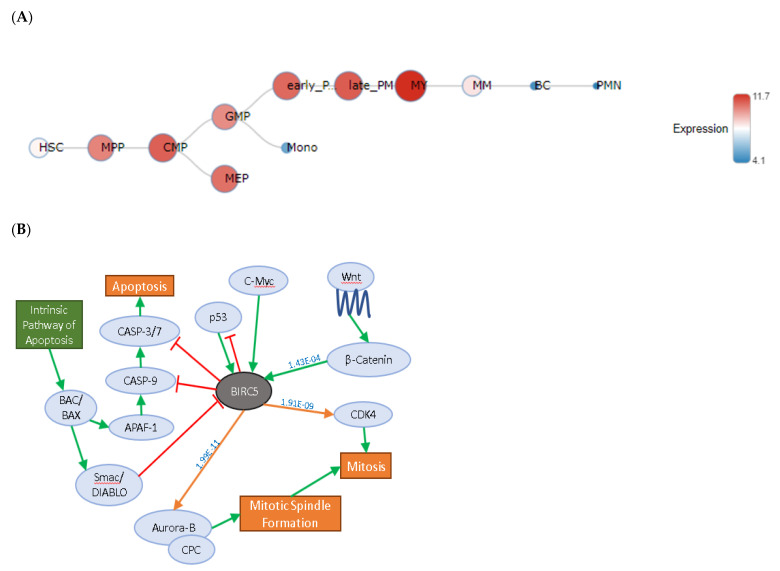
BIRC5 expression in health and disease. (**A**) *BIRC5* expression was analysed in healthy FACs sorted blood cells and analysed using the BloodSpot dataset (accessed on 27 March 2021; [20]). Expression was shown to be highest in myelocytes (MY), late promyelocytes (late_PM), early promyelocytes (early_PM), common myeloid progenitors (CMP), megakaryocyte/ erythroid precursor (MEP) and multipotent progenitors (MPP). No expression was detected in haematopoietic stem cells (HSC) and metamyelocytes (MM) with decreased expression in band cells (BC) and polymorphonuclear cells (PMN); (**B**) interactions between BIRC5 and other proteins in adult AML based on peer-reviewed published data (caspases [23,24,25]; *p53* [22,26,27]; C-Myc [28]; Wnt/β-catenin [29]; CDK4; [30]; mitotic spindle formation [31,32]; BAC/BAX/DIABLO [24,33]). Values above the arrows indicate the *p*-values of the relationships between the two gene probesets following analysis using the Microarray Innovations In Leukemia (MILE) dataset [34] (Table S1).

Using BloodSpot [20] we found that BIRC5 was predominantly expressed in the early promyelocyte lineage, common myeloid progenitors megakaryocyte/erythroid precursor (MEP) and multipotent progenitors (Figure 1A).

### 2.2. Pathway Analysis

We examined the pathways that BIRC5 engages in using in silico searches and RNA-seq data based on our own previous studies [19]. We found that BIRC5 directly engages with genes involved in pathways associated with the hallmarks of cancer [21] (Figure 1B; Table S1) including apoptosis and mitosis. 

BIRC5 is periodically expressed during the cell cycle, with weak expression in G1, multiplied by six in the S phase and by more than 40 in G_2_/M. During mitosis BIRC5 is involved in spindle assembly checkpoint and cytokinesis. BIRC5 is downregulated by p53 to allow apoptosis to occur and in this way regulates cell proliferation and cell death [22].

### 2.3. Gene Expression Analysis

#### 2.3.1. BIRC5 Expression and Clinical Features of AML

**Figure 2:**
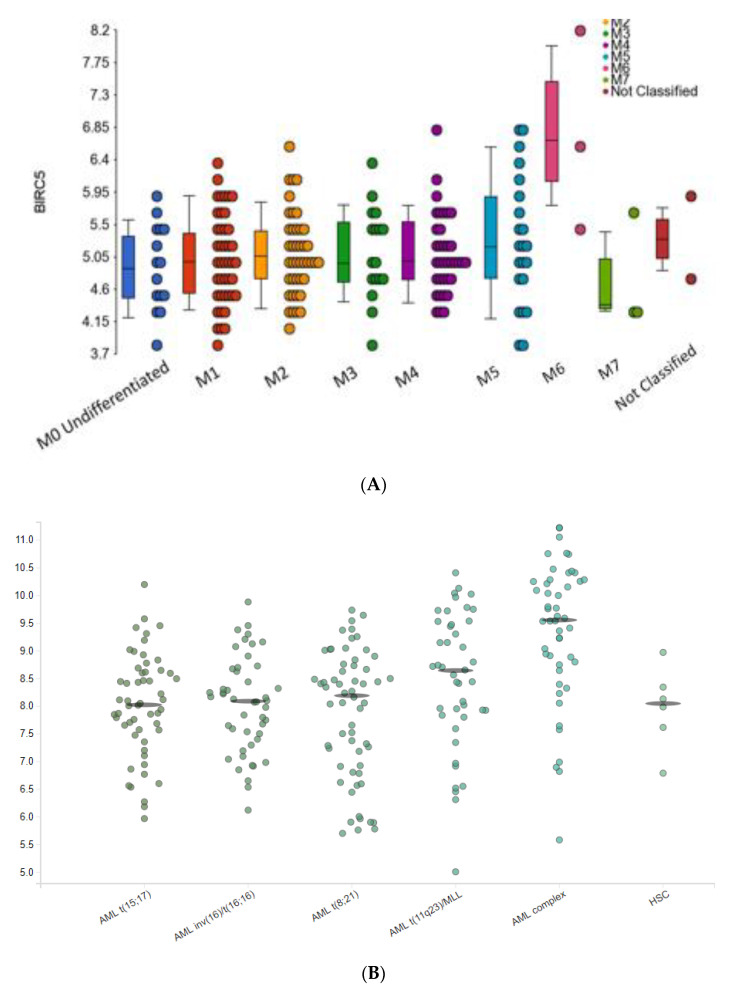
*BIRC5* expression in disease. *BIRC5* expression was (**A**) elevated in the FAB M6 subtype of AML and decreased in the M7 subtype in comparison to all other FAB subtypes although patient numbers in rare subgroups were small (*n* = 3 per group). *BIRC*5 expression was (**B**) elevated in patients with complex cytogenetic abnormalities when compared to all other cytogenetic groups in the BloodSpot dataset. Y-axis shows log2 expression in each graph.

**Figure 3:**
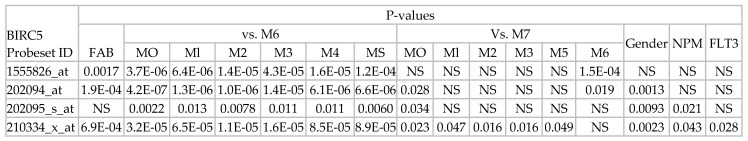
Association as indicated by *p*-values between BIRC5 and patient clinical features in adult AML (MILE; dataset A). NS: not significant.

Examination of the relationship between each *BIRC5* probesets and the clinical features of adult AML (MILE; dataset A) showed a significant difference in *BIRC5* expression between genders, Nucleophosmin 1 (NPM1) mutation and wild type (WT), M6 and all other French American British (FAB) subtypes (M0–M5, M7), and M7 and all other FAB subtypes (M0–M6) (Figure 2A and Figure 3). Analysis of *BIRC5* transcription showed elevated levels in M6 and decreased levels in the M7 FAB subtypes. M6 is also known as erythroleukaemia or Di Guelielmo Syndrome and is typified by the myeloprofileration of erythrocyte precursors while M7, also known as acute megakaryocytic leukaemia, accounts for only 1% of all adult AML cases and arises from immature platelet precursors, unlike the other FAB subtypes (M0–M5) which occur in immature leukocytes. In addition, patient numbers in the M6 and M7 FAB subgroups were very low (*n* = 3 each) (Figure 2A) making this data observationally interesting but in need of more patient numbers for verification.

#### 2.3.2. BIRC5 Expression Correlates with Poor Outcome Cytogenetics

BloodSpot data indicated that the highest expression of BIRC5 was in AML patients with complex cytogenetic abnormalities compared (Figure 2B) with all other cytogenetic abnormalities while MILE data indicated a correlation between higher levels of BIRC5 and poor prognosis cytogenetics (*p* = 0.02).

#### 2.3.3. BIRC5 Expression Correlates with Genes Involved in Cell Cycle

**Figure 4:**
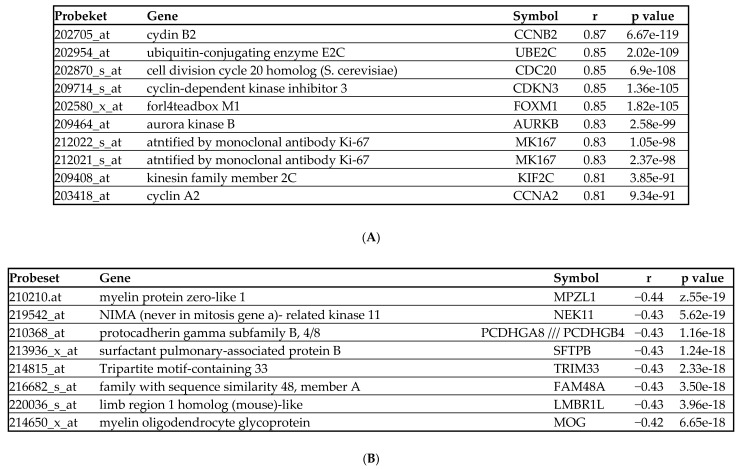
Genes with the greatest (**A**) positive and (**B**) negative correlation with *BIRC*5 expression (202095_s_at).

**Figure 5:**
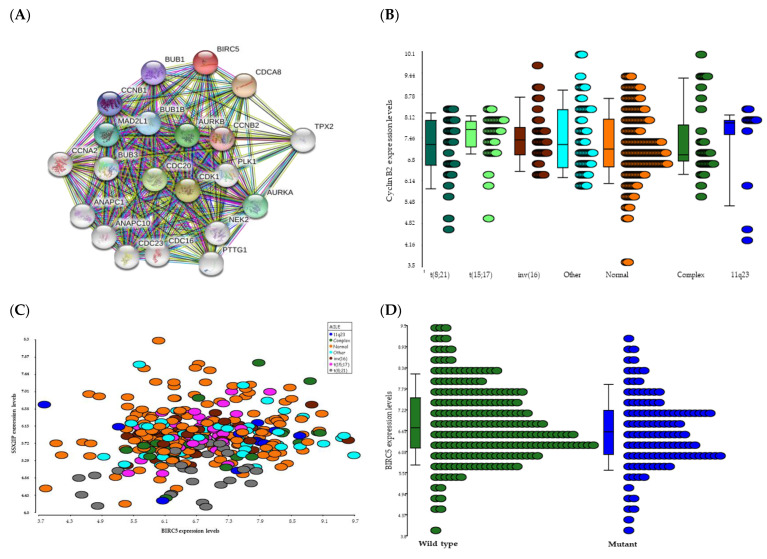
*BIRC*5 molecular interactions (**A**) STRING analysis indicated that BIRC5 was co-expressed with a number of cell cycle related proteins, including Cyclin B2 (CCNB2); (**B**) Cyclin B2 (202705_at) was found to be elevated in 11q23 patients (*p* = 0.002); (**C**) *SSX2IP* (203015_s_a5; y-axis) and *BIRC5* (202095_s_at; x-axis) expression were associated (r = 0.301); (**D**) *BIRC5* showed a correlation with WT (green balls) rather than mutated *FLT*3 (blue balls) in patients from the MILE study (*p* = 0.03). Probe 202095_s_at expression is shown in each panel but represents the results with each *BIRC5* probe.

Expression of *BIRC5* correlated with a number of genes involved in cell cycle regulation (Figure 4A) as demonstrated through gene expression analysis of the MILE data and using the search tool for recurring instances of neighbouring genes (STRING) analysis (Figure 5A). The highest correlation between *BIRC5* expression was with cyclin B2 (*CCNB*2) which showed elevated expression in AML patients with 11q23 and t(15;17) (Figure 5B). Inverse correlations were also found with myelin protein zero-like 1 expression (*MPZL*1), Never in mitosis gene a-related kinase 11 (*NEK*11) and protocadherin gamma subfamily B, 4/8 (*PCDHG*B4/A8) (Figure 4B). Although not in the top 10 associations shown, there was a close association between BIRC5 and SSX2IP (Figure 5C) expression. *SSX2IP* has previously been shown to be associated OS in AML patients that are cytogenetically normal (CN) [35], with cell cycle, and specifically *CDC*20 [36] and downregulated in t(8;21) patients [36].

#### 2.3.4. BIRC5 Expression Correlates with WT but Not Mutated FLT3

*BIRC*5 has previously been shown to mediate blast cell proliferation in mice with Flt3-ITD [37], however, in the MILE dataset, *BIRC*5 expression was found to show a correlation with WT rather than mutated *FLT*3 (Figure 5D).

#### 2.3.5. BIRC5 Is Associated with Disease/Relapse Free Survival, but Not OS, in Adults with AML

**Figure 6:**
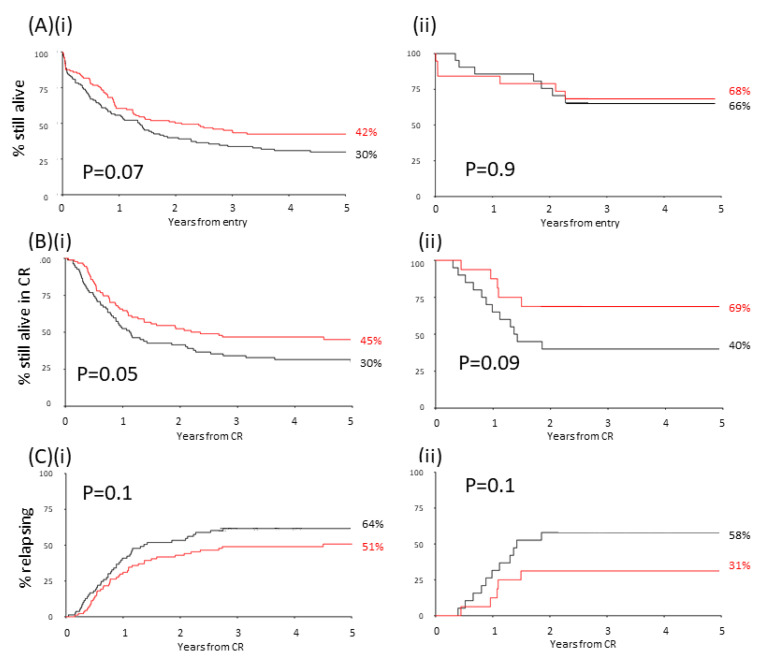
Above median levels of *BIRC5* is associated with increased OS rates and disease-free survival (MILE/dataset A). (**A**) There was a trend towards above median levels of *BIRC5* being associated with improved OS but this did not reach statistical significance (**i**) for the whole cohort or (**ii**) when examining CBF patients alone; (**B**) examination of disease free survival and its association with above and below median *BIRC5* levels reached significance when examining (**i**) the whole MILE dataset but (**ii**) was not indicated when examining CBF patients alone; (**C**) there was no association between above or below *BIRC5* levels and relapse for either (**i**) the whole MILE dataset or (**ii**) CBF patients alone. *p*-values from Log-Rank analysis. Black line absent; red line present.

When examining the MILE data/dataset A there were no correlations between above and below median levels of BIRC5 and trial, age, sex, cytogenetics, performance status, secondary disease or white blood cell counts. However OS showed a trend with BIRC5 present calls (Figure 6Ai) while relapse/disease free survival was significantly associated with BIRC5 present calls (Figure 6Bi) although this association was not found in CBF patients when examined alone (Figure 6Aii,Bii).

In adjusted analyses neither level nor present calls associated with complete remission (CR) rates. However, there was no difference in BIRC5 levels between those patients who relapsed and those who did not (Figure 6Ci) even when examining the CBF group alone (Figure 6Cii). BIRC5 levels did not correlate with overall remission (OR) hazard ratio (HR) 0.85 (0.52–1.37) *p* = 0.5 and similarly BIRC5 present calls did not correlate with OR, 0.74 (range 0.39–1.40) *p* = 0.4 in the whole cohort or when examining Core Binding Factor (CBF) patients alone, 1.09 (range 0.05–22.45), *p* = 0.9.

There was some evidence of a beneficial effect, in adjusted analyses, where above median levels of BIRC5 were shown to be associated with OS 0.65 (0.51–0.84) *p* = 0.001 which was maintained when CBF patients were analysed alone HR 0.16 (0.03–0.90) *p* = 0.03. There was a beneficial effect, in adjusted analyses, between above median BIRC5 levels and relapse free survival HR 0.51 (0.37–0.70) *p* < 0.0001 which was not significant when the CBF patients were examined alone 0.30 (0.08–1.20) *p* = 0.08. 

Below median levels of BIRC5 were associated with elevated relapse rates in AML patients in adjusted analyses HR 0.54 (0.38–0.76) *p* = 0.0005 but this was not observed maintained when the CBF group of AML patients were analysed alone HR 0.37 (0.09–1.49) *p* = 0.15.

**Figure 7:**
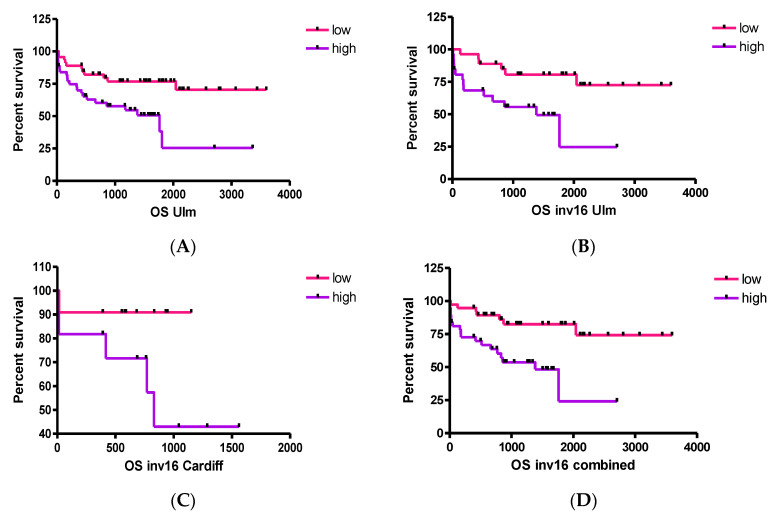
Correlation of *BIRC5* mRNA expression with OS in patients with CBF-AML. (**A**) Correlation of *BIRC5* mRNA expression levels with OS in 93 CBF cases (data set B, log-rank test, *p* = 0.004); (**B**,**C**) correlation of *BIRC5* expression with OS in 55 (data set B, Log-Rank test, *p* = 0.004) and 22 inv(16) cases, respectively (dataset A, Log-Rank test, *p* = 0.077); (**D**) correlation of *BIRC5* expression with OS in the combined inv(16) data set (data sets A and B, Log-Rank test, *p* = 0.001). The terms “high” or “low” *BIRC5* expression refer to an expression greater and lower than the median expression across all AML samples, respectively.

In order to further address the impact of *BIRC5* expression on AML patient survival, we evaluated its expression in a second independent microarray data set (referred to as the cDNA/data set B). This microarray data derived from AML cases comprising all cytogenetic AML subgroups [38] and again showed there was no correlation between *BIRC5* expression levels and age, BM blasts, lactate dehydrogenase (LDH), preceding malignancy or OS. However, we observed a correlation with distinct cytogenetic groups with significantly higher expression levels of *BIRC5* in AML cases with monosomy 7/loss of 7q or a t(15;17) (one-way analysis of variance, *p* < 0.001; data not shown). In 138 CN-AML cases [39] we found no significant correlation with the prognostically relevant genotype *NPM1*-mutated/*FLT3*-ITD-negative. However, in Core Binding Factor (CBF)-AML cases [40] lower *BIRC5* expression was associated with better clinical outcome (*p* = 0.004, Figure 7A). Notably, this was mainly due to inv(16) cases with low *BIRC5* expression (*p* = 0.007, Figure 7B). For AML cases with t(8;21) we found no significant difference (data not shown), despite the fact that BIRC5 seems to be a critical regulator of AML1/ETO-induced oncogenicity in AML [41]. In data set B we found an association between *BIRC5* and *FLT3* wild-type status (*p* = 0.041) and with days in remission (*p* = 0.028). In dataset A/MILE, high expression levels of *BIRC5* correlated with worse survival in inv(16) patients too (*p* = 0.077, Figure 7C) with highly significant findings when data from both studies were combined (*p* = 0.001, Figure 7D).

## 3. Discussion

BIRC5 has been shown to play essential roles in cell cycle progression and mitosis. It binds with the chromosomal passenger complex (CPC) and Aurora-B kinase in the nucleus, leading to correct mitotic spindle formation [32]. Conversely, BIRC5 depleted cells have been shown to exit mitosis with incorrect chromosomal alignment [31] and this is supported by the gene correlations between *BIRC*5 and other gene products involved in the formation of the mitotic spindles identified in this study. Although Bloodspot showed that the highest levels of BIRC5 were present in patients with complex karyotypic abnormalities, it also showed there was no significant difference in the levels of BIRC5 expression in haematopoeitic stem cells and patients with inv(16), t(8;21), 11q23 or t(15;17). We have previously shown that BIRC5 was associated with different 11q23/MLL abnormalities in adults with B-cell acute lymphocytic leukaemia [42] and in this study elevated BIRC5 expression was found in adult AML patients with complex cytogenetic abnormalities.

Our findings support data already generated in solid tumours showing a strong correlation between *BIRC5* expression and *AURKB*, *PLK1*, *TPX2*, *KIF2C* and *cyclin A2* expression [43,44,45]. Several clinical studies are ongoing with the therapeutic aim of inhibiting *AURKB* [46] in an effort to target genes involved in the “BIRC5 cancer network” and clinical responses indicate a central role of this pathway in proliferating leukaemic cells. Indeed, many of the genes that *BIRC5* has been shown to interact with are cell cycle related and for exemplification, the most significant association between *BIRC*5 and any other gene, in this study, was with Cyclin B2. Cyclin B2 has been shown to stimulate the proliferation of triple negative breast cancer cells [47] and to alter mitotic spindle checkpoint control leading to the genomic instability seen in cancer [48]. In addition, Cyclin B2 has been shown be an independent prognostic biomarker in invasive breast cancer [49]. Using selective siRNA-mediated silencing to decrease the expression of *BIRC5* has been shown to increase the sensitivity of colon epithelial cells to CDK inhibitors suggesting a mechanistic basis for the preclinical development of future CDK inhibitor-based therapeutic strategies [50]. 

We also found a correlation between increased *BIRC5* expression and FLT3 WT, a correlation that has not been identified in AML patient samples previously, with other studies of the interactions between *BIRC5* and *FLT*3-internal tandem duplication (ITD) being made predominantly through cell line studies and mouse models. For example other investigators have shown that BIRC5 mediates acute leukaemia in mice induced by Flt3-ITD [37] and that BIRC5 confers resistance to FLT3 inhibitors [51]. In addition, FLT3 inhibitors have been shown to cause anti-proliferative activity, in leukaemia cell lines with FLT3-ITD, through the downregulation of MCL-1 and BIRC5, the latter via the STAT3/5 pathway [52]. 

We have previously described the role of many leukaemia associated antigens (LAAs) in cell cycle [53] and the association between *BIRC5* and the expression of LAAs such as *Synovial Sarcoma X breakpoint 2 interacting protein* (*SSX2IP*) and *hyaluronan-mediated motility receptor* (*HMMR; RHAMM*) expression (*p* < 0.001) has been described [35,54,55]. Indeed, we have described a better outcome in AML patients co-expressing LAAs such as *HMMR*, *CA9*, *PRAME* and *SSX2IP* which are associated with cell proliferation in vitro [36,56]. 

CBF is a heterodimeric protein complex involved in the transcriptional regulation of normal haematopoiesis. Mutations in CBF-encoding genes (such as t(8;21) and inv(16)) result in leukaemia-associated proliferative advantages. CBF-AML accounts for around 20% of all AML patients and is often associated with improved outcomes compared to other subtypes of AML. However, it should be noted that although modern therapies may improve remission rates, they often lead to relapse, meaning the development of targeted therapies is still needed for improved outcomes. There was an association between decreased *BIRC5* expression and improved clinical outcomes due to inv(16) but this same association was not seen with t(8;21) patients despite AML-ETO being a critical regulator of BIRC5 in AML [41]. Although we did not find that above or below median expression of *BIRC5* correlated with OS, we did find that above median expression of *BIRC5* correlated with relapse-free survival (MILE dataset) and while inv(16) correlated with low *BIRC5* levels in the cDNA/Dataset B, the correlation between inv(16) and above median levels of *BIRC5* and poorer survival were more obvious following the combination of both datasets in this study. This may reflect the higher percentage of inv(16) patients in the CBF cohort in dataset B compared with dataset A.

BIRC5 has been targeted by immunotherapy in a number of ways (recently reviewed in [57]) including through combined treatment with YM155, a novel small molecule transcriptional inhibitor of *BIRC5* which when used with chemotherapeutic agents can increase drug efficacy on AML cells [58]. Although BIRC5 is an intracellular protein and therefore not a good target for CAR-T therapies, BIRC5-peptide mediated immunotherapy has been shown to exhibit low toxicity in clinical trial and can increase BIRC5 peptide-specific CTLs that kill cancer cells [59]. Alternatively genetically modified TCRs could be used to target BIRC5 expressing cancer cells [60], especially because of its wide overexpression in a number of tumour types including leukaemia and with regard to this study AML. 

In summary, *BIRC5* expression appears to be able to predict better outcomes at least in a subset of CBF-AML cases (those with inv(16)) suggesting that this LAA may provide an immunologically relevant personalised target for a sub-group of AML patients.

## 4. Materials and Methods

### 4.1. BIRC5 Expression in Healthy Haematopoietic Cells

The BloodSpot database [20] includes 23 high-quality curated data sets relevant to normal and malignant blood formation and, in addition, includes a unique integrated data set, called BloodPool. The effect expression had on the OS, was observed via the use of Bloodspot [20] an online microarray database containing expression and clinical data. The MILE study is a multi-laboratory database containing more than 3000 whole genome microarray analysis [34]. It was headed by the European Leukemia Network (ELN) and sponsored by Roche Molecular Systems, Inc. (Pleasanton, CA, USA).

### 4.2. BIRC5 Protein Interaction Analyses

Relationships between BIRC5 and other genes/proteins were established using Kyoto Encyclopedia of Genes and Genomes (KEGG; www.kegg.jp), STRING (https://string-db.org/ accessed on 27 March 2021 [61]), WEB-based Gene Set Analysis Toolkit and a comprehensive literature was performed searching for the interactions between proteins with BIRC5. Confirmation of the correlation between gene transcripts and *BIRC5* was determined using the MILE dataset (GSE13159). When multiple probe sets were available for *BIRC5* the following were used: 202094, 208052 and 212399.

### 4.3. Association between Genes and Clinical Features

Examination of the relationship between each BIRC5 probeset and the clinical features of adult AML was performed using data generated by the TCGA research network: http://www.cancer.gov/tcga (accessed on 27 March 2021). The association between BIRC5 levels in samples from patients with NPM mutation and WT, FLT3-ITD and FLT-WT, and all FAB subtypes were examined.

The MILE/Data set A comprised 312 AML samples including 180 CN-AML and 63 CBF samples including 31 cases with t(8;21) and 32 with inv(16). Samples were analysed using Affymetrix human genome U133A 2.0 or human genome U133 Plus 2.0 microarrays (Cardiff/MILE/data set A, [34]). cDNA/Dataset B comprised 306 AML samples including 168 CN cases and 93 CBF leukaemias including 38 cases with t(8;21) and 55 with inv(16), each analysed by 40k cDNA microarrays [39,40]. 

With regard to both datasets, gene expression profiling (GEP) was performed as previously described [38] using Affymetrix microarray technology in accordance with the manufacturer’s recommendations. Fluorescence ratios were normalised by applying the RMA Log2 values and any batch effect removed using Partek Genomics Suite (St Louis, MO, USA). In selected cases, BIRC5 GEP data was validated by quantitative RT-PCR as previously reported [56]. 

For the correlation with survival data, expression values were dichotomised by the median expression of the respective gene across all AML samples and statistical analyses were performed as described previously [38,56].

## 5. Conclusions

Analysis of independent AML datasets using different microarray platforms showed that in AML *BIRC5* mRNA expression is strongly associated with the expression of *AURKB*, *PLK1*, *TPX2*, *HMMR* and *SSX2IP* as well as other important cell cycle associated genes. Downregulation of this complex system involved in tumorigenesis might provide important targets for tumour cell control in acute leukaemias. We also showed that patients with CBF AML, and particularly patients with inv(16), who have above median levels of *BIRC5*, have poorer survival outcomes. This indicates that those AML patients with inv(16) would provide interesting candidates for immunotherapies that target BIRC5.

## Data Availability

All data has been made publicly available at the time of previous studies cited in the text, and accessed in this study as described. Raw data is also available from Figure 1B and Supplementary Table S1.

## Supplementary Materials

The following are available online at https://www.mdpi.com/article/10.3390/ijms221910482/s1, Table S1: Significance of the relationship between BIRC5 and the expression of other genes in AML patients (DOI:10.5281/zenodo.4923749).

## Abbreviations

AML	acute myeloid leukaemia
BIRC5	Baculoviral IAP Repeat Containing 5BM bone marrow
CBF	Core Binding Factor
CN	Cytogenetically normal
CR	Complete remission
FAB	French American British
FLT3	FMS-like tyrosine kinase 3
HR	Hazard Ratio
IAP	Inhibition of Apoptosis
ITD	internal tandem duplication
KEGG	Kyoto Encyclopedia of Genes and Genome
MILE	Microarray Innovations In Leukemia
NPM1	Nucleophosmin 1
OR	overall remission
OS	overall survival
RUNX1	RUNX Family Transcription Factor 1
STRING	search tool for recurring instances of neighbouring genes
TCGA	The Cancer Genome Atlas
WT	wild type
WT1	Wilm’s Tumour gene 1
